# Global and Local Optimization Algorithms for Optimal Signal Set Design

**DOI:** 10.6028/jres.106.019

**Published:** 2001-04-01

**Authors:** Anthony J. Kearsley

**Affiliations:** National Institute of Standards and Technology, Gaithersburg, MD 20899-8910

**Keywords:** Nonlinear programming, optimization, signal set

## Abstract

The problem of choosing an optimal signal set for non-Gaussian detection was reduced to a smooth inequality constrained mini-max nonlinear programming problem by Gockenbach and Kearsley. Here we consider the application of several optimization algorithms, both global and local, to this problem. The most promising results are obtained when special-purpose sequential quadratic programming (SQP) algorithms are embedded into stochastic global algorithms.

## 1. Introduction

**Fig. 1:**
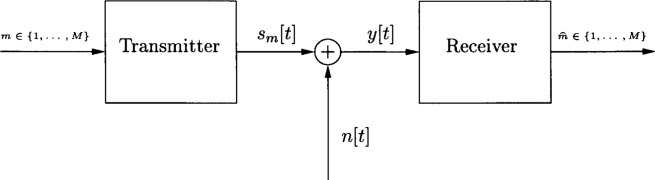
Model of a communication system.

Consider the simple communication system model shown in Fig. 1. The goal is to transmit one of *M* possible symbols, i.e., an *M*-ary signaling system, over a memoryless additive noise channel. We will assume all signals are discrete-time with *T* samples. The transmitter assigns a unique signal *s_m_* : {1, …, *T*} → ℝ to each symbol *m* ∈ {1, …, *M*}. It is this signal that is sent through the channel. At the other end, the received signal is

$$y[t]=s_{m}[t]+n[t],\;t=1,\ldots ,T,$$

where *n* : {1, …, *T*} → ℝ is a noise process, and the job of the receiver is to decide which symbol was transmitted. Our goal is to design a set of signals *s_m_*, *m* = 1, …, *M*, which maximize, subject to constraints on the signals, the probability of a correct decision by the receiver given a particular channel noise distribution.

Of course, in order to design an optimal signal set, the action of the channel and the receiver must be completely specified. For the channel, we assume the noise process is independent and identically distributed (iid) with distribution *p_N_*. Further, we assume that the noise process is independent of the symbol being transmitted. Our detection problem falls into the class of *M*-ary Bayesian hypothesis testing problems where, for *m* = 1, …, *M*, the hypotheses are defined as follows,

$$H_{m}:y[t]=s_{m}[t]+n[t],\;t=1,\ldots ,T.$$

To simplify notation, define the received signal vector

$$y≜{\left(y[1],\ldots ,y[T]\right)}^{T}.$$

Finally, it is assumed that the receiver was designed using the minimum average probability of error criterion (or the uniform cost criterion). It is well known that (see, e.g., Sec. IV.B of Ref. [20]), under our assumptions, the optimal receiver is the *maximum a posteriori probability* (MAP) detector. Specifically, the optimal receiver chooses

$$\hat{m}(y)=\arg\;\max\{p(H_{m}|y)|m=1,\ldots ,M\},$$

i.e., the hypothesis with the largest probability given the observation **y**.

Clearly, the receiver will make an error if hypothesis *H_m_* is true, but

$$p(H_{m^{\prime }}|y)>p(H_{m}|y),$$

for some *m'* ≠ *m*. Thus, the probability of a correct decision under hypothesis *H_m_* is

p({correctdecision}|Hm)=p({p(Hm|y)>p(Hm′|y),∀m′≠m}|Hm)=p({lnp(Hm|y)p(Hm′|y)>0,∀m′≠m}|Hm),

where, in order to put things in terms of the familiar log-likelihood ratio, we have assumed *p*(*H_m_*′|**y**) > 0 for all **y**, *m*′ ∈ {1, …, *M*}. For the signal set design problem considered here, no knowledge of the prior distribution on the hypotheses *H_m_*, *m* = 1, …, *M*, will be assumed. Of course, the conditional distribution *p*(*H_m_*|**y**) is known since, given a signal set, this distribution is completely determined by the distribution on the channel noise. Specifically, in view of our assumptions,

$$p(H_{m}|y)=\sum_{t=1}^{T}p_{N}(y[t]-s_{m}[t]).$$

If the prior distribution were known, the quantity to be maximized could be expanded as

$$p(\{\text{correct decision}\})=\sum_{m=1}p(\{\text{correct decision}\}|H_{m})\cdot p(H_{m}).$$

As *p*(*H_m_*) is not assumed to be known, the worst-case prior distribution will be used to compute *p*({correct decision}) for any given signal set. In particular, let

$$S≜\left\{\gamma \in \mathbb{R}|\sum_{m=1}\gamma_{m}=1,\;\gamma_{m}\geq 0,\;m=1,\ldots ,M\right\}.$$

The goal will be to find signal sets which maximize

$$\underset{\gamma \in S}{\min}\sum_{m=1}p(\{\text{correct decision}\}|H_{m})\cdot \gamma_{m}.$$

It is not difficult to show that this is equivalent to maximizing

$$\underset{m\in \{1,\ldots ,M\}}{\min}p(\{\text{correct decision}\}|H_{m}).$$ (1)

A standard assumption in transmitter design is that the signals are restricted to be of the form

$$s_{m}[t]≜\sum_{k=1}^{K}\alpha^{m,k}\phi_{k}[t],$$ (2)

where *ϕk* : {1, …, *T*} → ℝ, *k* = 1, …, *K*, are given basis functions and *α^m,k^* ∈ ℝ, *m* = 1, …, *M*, *k* = 1, …, *K*, are the free parameters. Finally, due to power limitations in the transmitter, the signals are forced to satisfy some type of power constraint, either peak amplitude or average energy. In this paper, we will assume a peak amplitude constraint, i.e.,

$$\left|s_{m}[t]\right|\leq C,\;m=1,\ldots ,M,\;t=1,\ldots ,T,$$ (3)

where *C* > 0 is given. Note that we could just as easily have considered an average energy constraint in our formulation. Our design problem is thus reduced to choosing parameters *α^m,k^* in order to maximize Eq. (1), subject to the constraints Eq. (3).

## 2. The Optimization Problem

The details of casting the design problem introduced in the previous section in such a way that it may be solved using standard nonlinear programming algorithms are presented in this section. The design of optimal signal sets under the assumption of Gaussian noise has been well studied (see, e.g., Ref. [22]). In fact, a gradient-based first-order algorithm was developed and analyzed in Ref. [5] for the case of Gaussian noise, *K* = 2 basis functions, and an average energy constraint on the signals. The performance of optimal detectors in the presence of non-Gaussian noise as a function of the signal set was first studied by Johnson and Orsak in Ref. [10]. It was shown in Ref. [10] that the dependence of detector performance on the signal set is related to the Kullback-Leibler (KL) distance between distributions for the various hypotheses. Based on this work, Gockenbach and Kearsley Ref. [7] proposed the nonlinear programming (NLP) formulation of the signal set design problem which is considered here.

Given our assumptions on the noise process, the log-likelihood ratio may be written

$$\ln\frac{p(H_{m}|y)}{p(H_{m^{\prime }}|y)}=\sum_{t=1}^{T}\ln\frac{p(H_{m}|y[t])}{p(H_{m^{\prime }}|y[t])}.$$

Note that, since randomness only enters the received signal through the additive noise process, when hypothesis *H_m_* is true, the receiver computes

$$p(H_{m}|y[t])=p_{N}(n[t]),$$

and, for *m*′ ≠ *m*,

$$p(H_{m^{\prime }}|y[t])=p_{N}(n[t]+(s_{m^{\prime }}[t]-s_{m}[t])).$$

Thus, upon substitution, the statistic of interest to us is

$$\ln\frac{p(H_{m}|y)}{p(H_{m^{\prime }}|y)}=\sum_{t=1}^{T}\ln\left(\frac{p_{N}(n[t])}{p_{N}(n[t]+(s_{m^{\prime }}[t]-s_{m}[t]))}\right).$$

Now, assuming the variance of the statistic (4) does not change as we vary *m*′ ≠ *m*, maximizing *p*({correct decision} | *H_m_*) is equivalent to maximizing the expected value of the statistic Eq. (4) for each *m*′ ≠ *m*. That is, under hypothesis *H_m_*, the probability of correctly choosing *H_m_* is maximized if we maximize

$$\underset{m^{\prime }\neq m}{\min}E\left\{\sum_{t=1}^{T}\ln\left(\frac{p_{N}(n[t])}{p_{N}(n[t]+(s_{m^{\prime }}[t]-s_{m}[t]))}\right)|H_{m}\right\}.$$

Thus, for the signal design problem considered here, we may equivalently use

$$\underset{m\in \{1,\ldots ,M\}}{\min}\underset{m^{\prime }\neq m}{\min}E\left\{\sum_{t=1}^{T}\ln\left(\frac{p_{N}(n[t])}{p_{N}(n[t]+(s_{m^{\prime }}[t]-s_{m}[t]))}\right)|H_{m}\right\}$$

as the objective function. Define the function *K_N_* : ℝ → ℝ as

$$K_{N}(\delta )≜\int_{\mathbb{R}}\ln\left(\frac{p_{N}(\tau )}{p_{N}(\tau +\delta )}\right)p_{N}(\tau )d\tau ,$$

i.e., the KL distance between the noise distribution and the noise distribution shifted by −δ. Note that if we assume a symmetric distribution for the noise (this is not a restrictive assumption), then *K_N_*(·) will be an even function. It is not difficult to show that

$$\begin{array}{c} E\left\{\sum_{t=1}^{T}\ln\left(\frac{p_{N}(n[t])}{p_{N}(n[t]+(s_{m^{\prime }}[t]-s_{m}[t]))}\right)|H_{m}\right\}\\ =\sum_{t=1}^{T}K_{N}(s_{m^{\prime }}[t]-s_{m}[t]). \end{array}$$

Define

$$\alpha ≜(\alpha^{1,1},\ldots ,\alpha^{1,K},\ldots ,\alpha^{M,1},\ldots ,\alpha^{M,K})\in {\mathbb{R}}^{MK}.$$

Substituting the expansion Eq. (2), we see that, under our assumptions, the signal set design problem is equivalent to solving the optimization problem

$$\begin{array}{l} \underset{\alpha \in {\mathbb{R}}^{MK}}{\min}\max\{-\sum_{t=1}^{T}K_{N}\left(\sum_{k=1}^{K}\left(\alpha^{m^{\prime },k}-\alpha^{m,k})\phi_{k}[t\right]\right)|m,m^{\prime }\\ \in \{1,\ldots ,M\},m^{\prime }>m\}\\ \text{s.t.}\;{\left(\sum_{k=1}^{K}\alpha^{m,k}\phi_{k}[t]\right)}^{2}\leq C^{2},m=1,\ldots ,M,t=1,\ldots ,T. \end{array}$$ (SS)

It is only necessary to consider *m*′ > *m* since *K_N_*(·) is an even function.

The problem (SS) is already in a form which may be handled by algorithms which tackle inequality constrained mini-max problems. Such algorithms have been developed by, e.g., Kiwiel in Ref. [13], Panier and Tits in Ref. [16], and Zhou in Ref. [25], all in the context of feasible iterates. In Ref. [25], Zhou extends the nonmonotone line search-based algorithm of Ref. [26] to handle the constrained case. The algorithm of Ref. [16] extends the feasible SQP algorithm of Ref. [17] to handle mini-max objective functions. A recent algorithm for the constrained mini-max problem which does not generate feasible iterates is the augmented Lagrangian approach of Rustem and Nguyen Ref. [23]. The problem may be converted into an equivalent single objective smooth nonlinear programming problem by adding an additional variable. This is the approach taken by Gockenbach and Kearsley in Ref. [7]. Additionally, they add a “regularization” term (possibly zero) to the converted objective. Specifically, consider

$$\begin{array}{l} \underset{\alpha \in {\mathbb{R}}^{MK},\gamma \in \mathbb{R}}{\min}-\gamma^{2}-\in_{r}{\left\|\alpha \right\|}_{2}^{2}\\ \;\text{s.t.}-\sum_{t=1}^{T}K_{N}\left(\sum_{k=1}^{K}\left(\alpha^{m^{\prime },k}-\alpha^{m,k})\phi_{k}[t\right]\right)\\ \;\leq -\gamma^{2},\;m^{\prime },m\in \{1,\ldots ,M\},\\ \;m^{\prime }>m,\\ {\left(\sum_{k=1}^{K}\alpha^{m,k}\phi_{k}[t]\right)}^{2}\leq C^{2},\;m=1,\ldots ,M,\;t=1,\ldots ,T,\\ \gamma \geq 0, \end{array}$$ (SS′)

where ***ϵ****_r_* is small (possibly zero).

It turns out that problems (SS) and (SS′) are difficult to solve using standard off-the-shelf nonlinear programming codes. To begin with, it was observed in Ref. [7] that outside of the feasible region, the linearized constraints for problem (SS) are often inconsistent, i.e. no feasible solution exists. This can be a big problem for sequential quadratic programming (SQP) based algorithms, generally considered the most successful algorithms available for NLP problems with a reasonable number of variables. Of course, with feasible iterates, the linearized constraints are always consistent and the solutions of the QP sub-problems are always well-defined. As an alternative to feasible iterates, there are infeasible SQP-based algorithms which use special techniques to deal with inconsistent QPs (see, e.g., Ref. [24, 12, 4]). Another difficulty in applying a local NLP algorithm is that problem (SS) has many local solutions which may prevent convergence to a *global* solution.

## 3. Local Algorithms

Sequential Quadratic Programming (SQP) has evolved into a broad classification encompassing a variety of algorithms. When the number of variables is not too large, SQP algorithms are widely acknowledged to be the most successful algorithms available for solving smooth nonlinear programming problems. For an excellent recent survey of SQP algorithms, and the theory behind them, see Ref. [4].

In general, an SQP algorithm is characterized as one in which a quadratic model of the problem is formed at the current estimate of the solution and is solved in order to construct the next estimate of the solution. Typically, in order to ensure global convergence, a suitable *merit function* is used to perform a line search in the direction provided by the solution of the quadratic model. While such algorithms are potentially very fast, the local rate of convergence is critically dependent upon the type of second order information utilized in the quadratic model as well as the method by which this information is updated.

### 3.1 Infeasible SQP

Numerous SQP algorithms that do not require iterates to remain feasible have been suggested by researchers (e.g., Refs. [3, 6, 24] among others). Because of the nonlinear nature of the constaints appearing in this specific class of problems, the linearizations employed by SQP are frequently inconsistent. As a result, constraint perturbation techniques must be employed to ensure that the algorithm can generate a descent direction at every iteration. This, at least in part, explains the superior performance of hybrid SQP algorithms reported on here (see Tables 2 and 3). These algorithms are particularly desirable because they do not require a feasible starting point.

### 3.2 Feasible SQP

In Ref. [19, 17, 18, 9], variations on the standard SQP iteration are proposed which generate iterates lying within the feasible set. Such methods are sometimes referred to as “Feasible SQP” (or FSQP) algorithms. It was observed that requiring feasible iterates has both algorithmic and application-oriented advantages. Algorithmically, feasible iterates are desirable because

- The Quadratic Programming (QP) subproblems are always consistent, i.e., a feasible solution always exists, and
- The objective function may be used directly as a merit function in the line search.

State of the art SQP algorithms typically include complex schemes to deal with inconsistent QPs. Further, the choice of an appropriate merit function (to enforce global convergence) is not always clear. Requiring feasible iterates eliminates these issues. In order to overcome the problem of inconsistent QPs in this work, we use the CFSQP implementation Ref. [14] of the FSQP algorithm proposed and analyzed in Ref. [18] as our local algorithm.

## 4. Global Solutions

### 4.1 Stochastic Approach

In one attempt to overcome the problem of local solutions, we use a stochastic two-phase method (see, e.g., Ref. [1]) where random initial points are generated in the feasible region and a local method is repeatedly applied to a subset of these points. Such an approach may be thought of as simply a “smart” way of generating many initial points for our fast local algorithm with the hopes of eventually identifying a global solution. Specifically, we will use the Multi-Level Single Linkage (**MLSL**) approach Ref. [1, 11], which is known to find, with probability one, all local minima (hence the global minima) in a finite number of iterations.

Let ℳ denote the cumulative set of local minimizers identified by the **MLSL** algorithm. At iteration *ℓ*, for some integer *N* > 0 fixed, we generate *N* points $\alpha_{(\ell -1)N+1},\ldots ,\alpha_{\ell N}$ distributed uniformly over the feasible set *X* for (SS). For each of the points $\alpha_{i}\in \{\alpha_{1},\ldots ,\alpha_{\ell N}\}$ we check to see if there is another point α*_j_* within a “critical distance” *r_ℓ_* of *α_i_* which also has a smaller objective value. If not, then the local algorithm, call it ℒ is applied with initial point α*_i_* and the computed local minimizer is added to the set ℳ. After all points are checked, *r*_ℓ_ is updated, *ℓ* is set to *ℓ* + 1 and the process is repeated. At any given iteration, the local maximizer with the smallest objective value is our current estimate of the global solution. For ease of notation, define the (mini-max) objective

$$\begin{array}{l} F(\alpha )≜\max\{-\sum_{t=1}^{T}K_{N}\left(\sum_{k=1}^{K}\left(\alpha^{m^{\prime },k}-\alpha^{m,k})\phi_{k}[t\right]\right)|m,m^{\prime }\\ \in \{1,\ldots ,M\},m^{\prime }>m\}. \end{array}$$

Further, let ℒ(α) denote the local minimizer obtained when a local algorithm is applied to problem (SS) with initial point *α*. The following algorithm statement is adapted from Ref. [1].

#### Algorithm MLSL

*Step 0*. **set** ℓ ← 1, ℳ←Ø.
*Step 1*. **generate** *N* points *α*_(_*_ℓ_*_−1)_*_N_*_+1_, …, *α_ℓN_* uniformly over *X*.
   **set** *i* ← 1.
*Step 2*. **if** $\left(\exists j\;\text{s.t.}\;F(\alpha_{j})<F(\alpha_{i})\;\text{and}\;{\left\|\alpha_{i}-\alpha_{j}\right\|}_{s}<r_{\ell }\right)$ **then goto** *Step 3*.
   **else set** $ℳ\leftarrow ℳ\cup \{ℒ(\alpha_{i})\}$.
*Step 3*. **set** *i* ← *i* + 1.
   **if** *i* ≤ *ℓN* **then goto** *Step 2*.
   **else set** *ℓ* ← *ℓ* + 1 and **goto** *Step 1*.

It remains to specify how we select the critical distance *r_ℓ_*, the definition of the metric ||·||*_s_* we use for signal sets (as parameterized by α), and how we generate the sample points. Following Ref. [1], we use

$$r_{\ell }=\frac{1}{\sqrt{\pi }}{\left(\Gamma (1+n/2)\cdot m(X)\cdot \frac{\zeta \ln(\ell N)}{\ell N}\right)}^{1/n},$$

where *n* is the number of variables (*MK* for our problem), *m*(*X*) is the volume of the feasible region, and ζ > 2. To compute *m*(*X*), note that in view of symmetry with respect to the signals,

m(X)=A,

where *A* is the volume of the feasible region for the parameters corresponding to one signal (recall, *M* is the number of signals). The quantity *A* is easily estimated using a Monte Carlo technique.

Note that, for our problem, as far as the transmitter is concerned, a given signal set is unchanged if we were to swap the coefficients *α^m^*_1_^,^*^k^*, *k* = 1, …, *K*, with *α^m^*_2_^,1^, *k* = 1, …, *K*, where *m*_1_ ≠ *m*_2_. The distance “metric” we use in Algorithm **MLSL** should take this symmetry into account. Consider the following procedure for computing the distance between signal sets parameterized by α_1_ and α_2_.

**Table:** 

**function** dist(α_1_, α_2_) {
**for** *i* = 1, …, *M* − 1 **do** {
$d_{i}=\min\left\{\sum_{k=1}^{K}{\left(\alpha_{1}^{i,k}-\alpha_{2}^{j,k}\right)}^{2}|j\in \{1,\ldots ,M\}/\{j_{1},\ldots ,j_{i-1}\}\right\}$
$j_{i}=\text{arg}\;\text{min}\left\{\sum_{k=1}^{K}{\left(\alpha_{1}^{i.k}-\alpha_{2}^{j,k}\right)}^{2}|j\in \{1,\ldots ,M\}/\{j_{1},\ldots ,j_{i-1}\}\right\}$
}
$\text{return}{\left(\sum_{i=1}d_{i}\right)}^{1/2}$
}

This is not a metric in the strict sense of the definition, though it suffices for our purposes and we will set

$${\left\|\alpha_{1}-\alpha_{2}\right\|}_{s}≜\text{dist}(\alpha_{1},\alpha_{2}).$$

To aid the generation of sample points, before starting the **MLSL** loop we compute the smallest box which contains the feasible set *X*. By symmetry with respect to the signals, we can do this by solving 2*K* linear programs. Specifically, let ${\bar{\alpha }}^{k}\in \mathbb{R},k=1,\ldots ,K$ be defined as the optimal value of the linear program (LP)

$$\begin{array}{l} \underset{\alpha^{1,1},\ldots ,\alpha^{1,K}}{\max}\alpha^{1,k}\\ \text{s.t.}\;\sum_{q=1}^{K}\alpha^{1,q}\phi_{k}[t]\leq C,\;t=1,\ldots ,T,\\ \;\sum_{q=1}^{K}\alpha^{1,q}\phi_{k}[t]\geq -C,\;t=1,\ldots ,T, \end{array}$$ (U_k_)

and let ${\underline{\alpha }}^{k}\in \mathbb{R},k=1,\ldots ,K$ be defined as the optimal value of the LP

$$\begin{array}{l} \underset{\alpha^{1,1},\ldots ,\alpha^{1,K}}{\min}\alpha^{1,k}\\ \text{s.t.}\;\sum_{q=1}^{K}\alpha^{1,q}\phi_{k}[t]\leq C,\;t=1,\ldots ,T,\\ \;\sum_{q=1}^{K}\alpha^{1,q}\phi_{k}[t]\geq -C,\;t=1,\ldots ,T. \end{array}$$ (L_k_)

Then, it should be clear that

$$\begin{array}{l} X\subseteq B≜\{\alpha \in {\mathbb{R}}^{MK}|\alpha^{\text{m,k}}\in [{\underline{\alpha }}^{k},{\bar{\alpha }}^{k}],\\ k=1,\ldots ,K,m=1,\ldots ,M\}. \end{array}$$

Using standard random number generators, it is a simple matter to choose samples from the uniform distribution on the box B. Thus, for *Step 1* of Algorithm **MLSL**, we repeatedly generate samples from the uniform distribution on B, discarding those which do not lie in *X*, until we find *N* which do lie in *X*. It should be clear that such a procedure is equivalent to drawing *N* samples from the uniform distribution on *X*.

### 4.2 Expanded Space Approach

In this section we describe a reformulation of the problem along the lines of that considered in Ref. [8] in the context of molecular conformation. The essence of the approach is to add variables in such a way that the global solution of the new equivalent problem has an enlarged basin of attraction. While the method does not guarantee convergence to a global solution, it does increase the likelihood.

To use such an approach in our context, we assign to each signal *M* − 1 sets of “auxiliary” coefficients. Each set will be used exclusively for computing the interaction with one particular other signal. For a given signal, the method will asymptotically force these sets of auxiliary coefficients to coalesce, as they must in order for the solution to be feasible for the original problem. Let the auxiliary coefficients be denoted ${\tilde{\alpha }}^{m,\ell ,k}$, where *m*∈{1, …, *M*}, *ℓ*∈{1, …, *M*}/{*m*}, and *k*∈{1, …, *K*}. Thus, the problem has grown from *KM* to *KM*(*M* − 1) variables. Let $\tilde{\alpha }$ denote the vector of all such coefficients. As mentioned above, the coefficients ${\tilde{\alpha }}^{m,\ell ,k}$ and ${\tilde{\alpha }}^{\ell ,m,k},k=1,\ldots ,K$ will be used only to compute the interaction between signals *m* and *ℓ*. Correspondingly, define the objective function which encapsulates this interaction as

$$\kappa_{m,\ell }(\tilde{\alpha })≜-\sum_{t=1}^{T}K_{N}\left(\sum_{k=1}^{K}\left({\tilde{\alpha }}^{m,\ell ,k}-{\tilde{\alpha }}^{\ell ,m,k})\phi_{k}[t\right]\right).$$

**Fig. 2:**
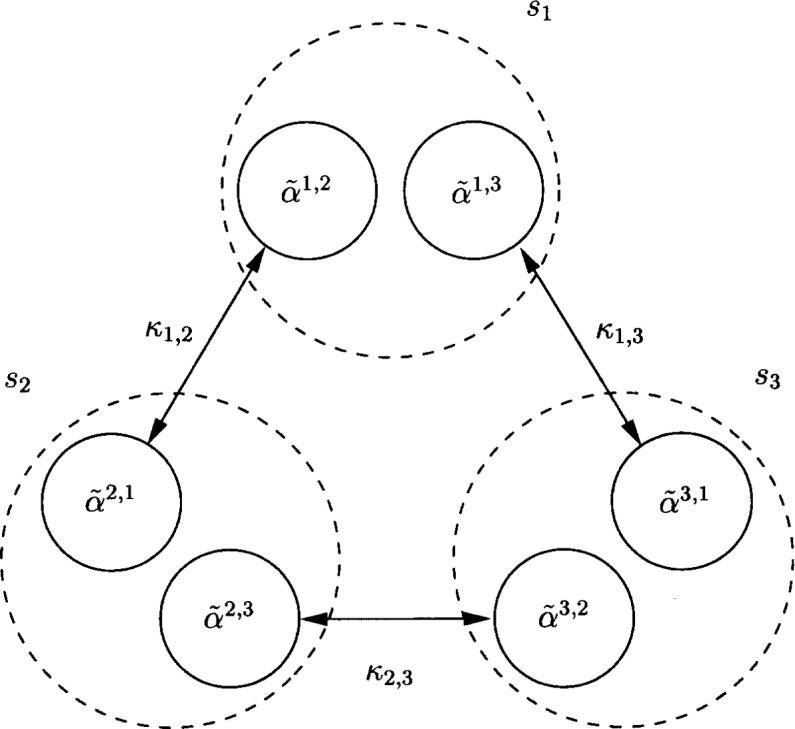
Schematic representation of auxiliary coefficient interactions.

A schematic representation of the auxiliary coefficients and their interactions is given in Fig. 2 for the case *M* = 3.

The constraint that the sets of auxiliary coefficients for each signal must all be equal at a feasible solution is easily expressed as a set of linear equality constraints. In particular, it is not difficult to show that there exists a linear operator *W*, a *KM*(*M* − 2) × *KM*(*M* − 1) matrix, whose kernel is precisely the set of all vectors satisfying this constraint. Finally, in view of this constraint, it is only necessary to enforce the power constraint on one set of auxiliary coefficients for each signal. Thus, our “equivalent” expanded space problem is

$$\begin{array}{l} \underset{\tilde{\alpha }\in {\mathbb{R}}^{KM(M-1)}}{\min}\max\{\kappa_{m,m^{\prime }}(\tilde{\alpha })|m,m^{\prime }\in \{1,\ldots ,M\},m^{\prime }>m\}\\ \text{s.t.}\;{\left(\sum_{k=1}^{K}{\tilde{\alpha }}^{1,2,k}\phi_{k}[t]\right)}^{2}\leq C^{2},\;t=1,\ldots ,T,\\ {\left(\sum_{k=1}^{K}{\tilde{\alpha }}^{m,1,k}\phi_{k}[t]\right)}^{2}\leq C^{2},\;t=1,\ldots ,T,\;m=2,\ldots ,M,\\ \;W\tilde{\alpha }=0. \end{array}$$ (ES)

Following Ref. [8], instead of attempting to solve this problem directly, we incorporate the equality constraints into the objective functions as a quadratic penalty term. This allows us to approach solutions from “infeasible” points by carefully increasing the penalty parameter and asymptotically forcing the auxiliary coefficients to coalesce for each signal. Specifically, define the penalized objective

$$P_{m,m^{\prime }}(\tilde{\alpha };\rho )≜\kappa_{m,m^{\prime }}(\tilde{\alpha })+\frac{1}{2}\rho^{2}{\tilde{\alpha }}^{T}W^{T}W\tilde{\alpha },$$

for *m*, *m*′ ∈ {1, …, *M*}, *m*′ > *m*. For a fixed value of *ρ* > 0, we can solve (using, e.g., a mini-max SQP-type algorithm) the problem

$$\begin{array}{l} \underset{\tilde{\alpha }\in {\mathbb{R}}^{KM(M-1)}}{\min}\max\{P_{m,m^{\prime }}(\tilde{\alpha };\rho )|m,m^{\prime }\in \{1,\ldots ,M\},m^{\prime }>m\}\\ \text{s.t.}\;{\left(\sum_{k=1}^{K}{\tilde{\alpha }}^{1,2,k}\phi_{k}[t]\right)}^{2}\leq C^{2},\;t=1,\ldots ,T,\\ {\left(\sum_{k=1}^{K}{\tilde{\alpha }}^{m,1,k}\phi_{k}[t]\right)}^{2}\leq C^{2},\;t=1,\ldots ,T,\;m=2,\ldots ,M. \end{array}$$ PES(ρ)

Let $ℒ({\tilde{\alpha }}^{0},\rho )$ denote the solution of *PES*(*ρ*) obtained using a local algorithm (such as those discussed in Sec. 3) starting with the initial point ${\tilde{\alpha }}^{0}$. Using the solution just obtained as the next initial point, the process may be repeated after appropriately increasing the penalty parameter *ρ*. At any time, a candidate global solution for the original problem (SS) may be computed by “projecting” a solution $\tilde{\alpha }=\tilde{ℒ}({\tilde{\alpha }}^{\prime },\rho )$ of *PES*(*ρ*) into ℝ*^MK^* and solving (SS) using a local algorithm with the projected vector as the initial point. We will denote the projection operation $\text{Proj}(\tilde{\alpha })$ and, using the notation from Sec. 4.1, the computed local solution for (SS) starting from the initial point $\text{Proj}(\tilde{\alpha })$ will be denoted $ℒ(\text{Proj}(\tilde{\alpha }))$. One possible method to compute the required projection is to simply take the arithmetic average of the corresponding components of the auxiliary coefficients, i.e., if $\alpha =\text{Proj}(\tilde{\alpha })$, then component wise

$$\alpha^{m,k}=\frac{1}{M-1}\sum_{\ell \neq m}{\tilde{\alpha }}^{m,\ell ,k},$$

*m* = 1, …, *M*, *k* = 1, …, *K*.

It remains to specify how we update the penalty parameter *ρ*. At “major” iteration^1^ *i*, the penalty parameter will be increased by a multiplicative factor, call it *δ_i_*, i.e.,

$$\rho_{i+1}=\delta_{i}\cdot \rho_{i}.$$

In addition, we will decrease the factor *δ_i_* when the projection for the current major iteration produces an estimate which does not improve upon the previous estimate. If *ρ_i_* is increased too fast for a given problem we could get trapped in a local solution. The precise algorithm statement follows.

#### Algorithm ES

$$\begin{array}{l} Data.\;{\tilde{\alpha }}_{0}\in {\mathbb{R}}^{KM(M-1)},\rho_{1}>0,\delta_{0}>1.\\ Parameters.\;\epsilon >0. \end{array}$$

*Step 0*. **set** *i* ← 1, *α*_0_ ← Proj( ${\tilde{\alpha }}_{0}$).
*Step 1*. **compute** ${\tilde{\alpha }}_{i}=\tilde{L}({\tilde{\alpha }}_{i-1},\rho_{i})$.
*Step 2*. **compute** $\alpha_{i}=L(\text{Proj}({\tilde{\alpha }}_{i}))$.
*Step 3*. **if** (*F*(*α_i_*) > *F*(*α_i_*_−1_) +*ϵ*) **then set** *α_i_* ← *α_i_*_−1_, ${\tilde{\alpha }}_{i}\leftarrow {\tilde{\alpha }}_{i-1}$, *ρ_i_* ← *ρ_i_*_−1_, $\delta_{i}\leftarrow \sqrt{\delta_{i-1}}$.
   **else set** *δ_i_* ← *δ_i_*_−1_.
*Step 4*. **set** *ρ_i_*_+1_ ← *δ_i_* · *ρ_i_*, *i* ← *i* + 1.
*Step 5*. **goto** *Step 1*.

### 4.3 Homotopy Approach

Suppose that for a given set of basis functions (and fixed problem size) we know the globally optimal signal set for one particular noise distribution, say *p*_1_(·). For example, in many cases it is possible to analytically compute the globally optimal signal set for Gaussian noise. In this section, we discuss a method for computing candidate globally optimal signal sets for a different noise distribution, say *p*_2_(·), based on this knowledge.

The so-called homotopy approach relies upon the observation that, for *λ* ∈ [0,1],

$$p_{N}(\tau ;\lambda )=(1-\lambda )p_{1}(\tau )+\lambda p_{2}(\tau )$$

is a valid noise distribution. Gradually increasing *λ* and repeatedly applying a local algorithm, the computed optimal signal set should trace out a continuous “path” from that for *p*_1_ to that for *p*_2_. Let *α*(*λ*, *α*_0_) denote the computed optimal solution of (SS) obtained via a local algorithm starting with the initial point *α*_0_ and using the noise distribution *p*_N_ (·;*λ*). At iteration *i*, we compute

$$\alpha_{i+1}=\alpha (\lambda_{i},\alpha_{i}),$$

and the homotopy parameter *λ_i_* is updated. The proper updating of *λ_i_* is critical. Clearly, we should have

$$\underset{i\to \infty }{\lim}\lambda_{i}=1.$$

Further, the rate of convergence of the method is directly related to how quickly *λ_i_* is increased. On the other hand, if *λ_i_* is increased too quickly then *α_i_*_+1_ may “jump” to a new path of minimizers. For preliminary tests we simply increment *λ_i_* by a small, fixed, amount.

#### Algorithm HOM

*Data*. *α*_0_ ∈ ℝ*^KM^*, 0 < *δ_λ_* << 1.

*Step 0*. **set** *i* ← 1, *λ*_0_ ← *δ_λ_*.
*Step 1*. **compute** *α_i_* ← *α*(*λ_i_*_−1_, *α_i_*_−1_).
*Step 2*. **if** *λ_i_* = 1 **then stop**.
*Step 3*. **set** *λ_i_*_+1_ ← min{*λ_i_* + *δ_λ_*, 1}.
*Step 4*. **set** *i* ← *i* + 1 and **goto** *Step 1*.

Numerically, one of the biggest challenges associated with this approach is the computation of the KL distance *K_N_* and the derivative of the KL distance ${K^{\prime }}_{N}$. For the distributions considered in this work these functions are readily obtained analytically. Unfortunately, this is no longer possible for convex combinations of these distributions as considered in this section. Consequently, we are forced to turn to numerical quadrature. For the preliminary numerical implementations we use a simple infinite trapezoid rule, i.e. we use the approximation

$$\int_{-\infty }^{\infty }f(\tau )d\tau \approx \frac{1}{\sqrt{N}}\sum_{k=-N}^{N}f\left(\frac{k}{\sqrt{N}}\right).$$

For those integrands with a slowly decaying tail we use the change of variables

$$\tau (t)=e^{t}-e^{-t}.$$

## 5. Numerical Results

Following Ref. [7], we consider the noise distributions *p_N_* listed in Table 1. For the definition of the Generalized Gaussian distribution, the constant *a* is defined as

$$a≜{\left(\frac{\sigma^{2}\Gamma (1/4)}{\Gamma (3/4)}\right)}^{1/2}.$$

For our numerical experiments, we assume *σ* = 1. The case *K* = 2 is of common interest, and we use either a sine-sine basis

$$\left\{\sqrt{\frac{2}{T}}\sin(2\pi \omega_{1}t/T),\;\sqrt{\frac{2}{T}}\sin(2\pi \omega_{2}t/T)\right\},$$

or a sine-cosine basis

$$\left\{\sqrt{\frac{2}{T}}\sin(2\pi \omega_{1}t/T),\;\sqrt{\frac{2}{T}}\cos(2\pi \omega_{1}t/T)\right\},$$

where *ω*_1_ = 10 and *ω*_2_ = 11. When *K* = 2 we can display the results in the plane as familiar signal *constellations*. Finally, we run experiments for *M* = 8, 16 signals, *T* = 50 samples, and with an amplitude bound of $C=\sqrt{10}$. Note that, for *M* = 16, problem (SS) has 32 variables, 120 objective functions, and 800 constraints.

In order to demonstrate the need for special-purpose SQP codes, we attempted to solve the problem using **VF02AD**^2^ from the Harwell subroutine library Ref. [15], a standard SQP code based on Powell’s algorithm Ref. [21] and a hybrid SQP code recently developed Ref. [3] and analyzed Ref. [2]. These codes do not directly solve mini-max problems, we used the formulation (SS′) suggested in Ref. [7]. In Tables 2 and 3, we list the number of times **VF02AD** and the *BKT* SQP algorithm Ref. [3] successfully converged to a local minimizer out of 20 trials for a given noise distribution and basis (and regularization parameter). In parenthesis we report on the number of times each algorithm succeeded in converging to the global minimizer. For each trial the initial point was drawn from the uniform distribution over the feasible set.

It is clear from the table that the standard SQP algorithm had little success converging to a local solution. The failures were essentially always due to inconsistent constraints in the QP sub-problem. In our trials, neither the Feasible SQP algorithm nor the hybrid *BKT* SQP algorithm failed to converge to, at least, a local solution.

**Fig. 3:**
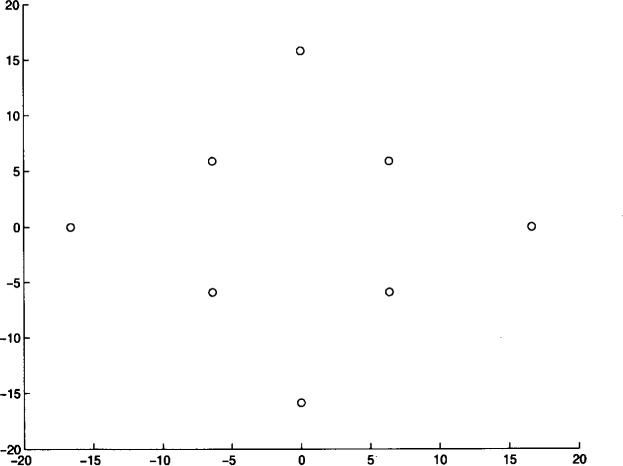
Optimal constellation for Gaussian noise, *M* = 8, sine-sine basis.

We ran Algorithm **MLSL** for 20 different problem instances. The algorithm was stopped after it appeared that no better local minimizers would be found (i.e., the estimate of the global minimum remained constant for several **MLSL** iterations). In Tables 4 and 5 we list our computed minimum values for instances of (SS) with *M* = 8 and *M* = 16, respectively. Note that our solutions respectively. Note that our solutions agree with those reported in Ref. [7]. In all cases, execution was terminated after no more than 10 to 15 minutes. In Figs. 3 through 8 we show the optimal signal constellations for several of the instances of (SS) corresponding to the optimal values listed in Tables 4 and 5.

## 6. Conclusions

In this paper we have presented an important and difficult optimization problem along with a broad arsenal of numerical optimization algorithms and modern enhancements that can be used to solve it. These problems are not “large-scale” by modern computing standards but they are, nonetheless, extremely difficult problems to solve efficiently.

Numerical solutions to these problems were located using SQP methods embedded into stochastic global algorithms. Numerous numerical tests suggest that this embedding procedure can significantly improve the performance of the SQP method.

Because there are so many different algorithms and implementations for the solution of nonlinear programming problems there is a need to create an accepted collection of test problems arising from the application of optimization. Because of the difficulties it poses, this family of problems is a natural candidate to use as a practical and significant test problem.

## Figures and Tables

**Fig. 4:**
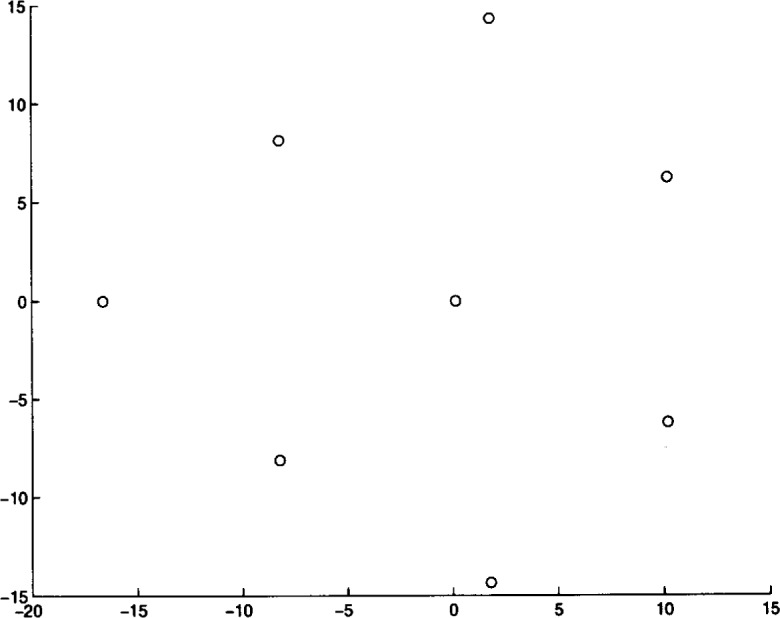
Optimal constellation for Generalized Gaussian noise, *M* = 8, sine-sine basis.

**Fig. 5:**
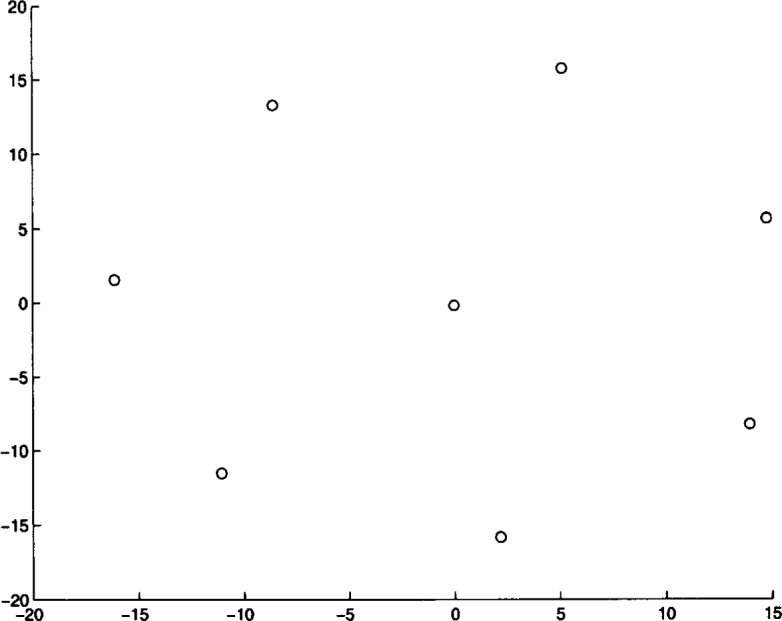
Optimal constellation for Laplacian noise, *M* = 8, sine-cosine basis.

**Fig. 6:**
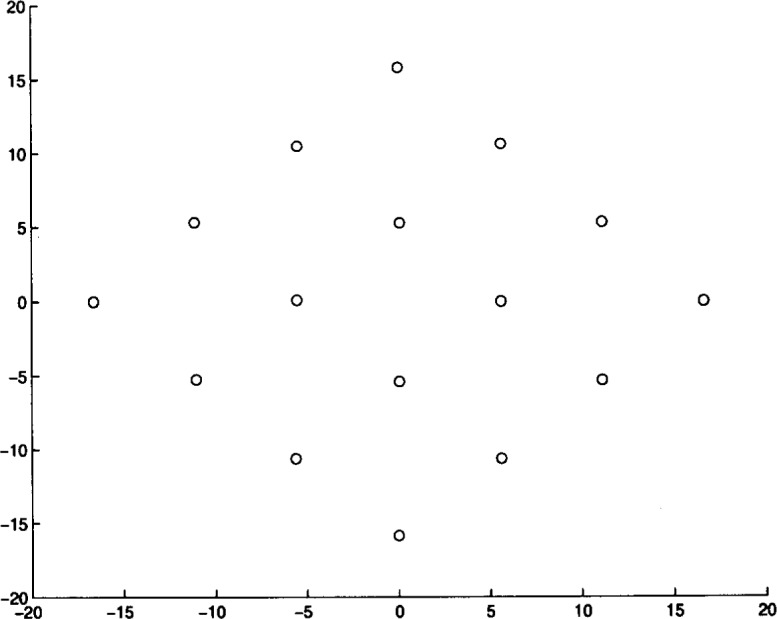
Optimal constellation for Cauchy noise, *M* = 16, sine-sine basis.

**Fig. 7:**
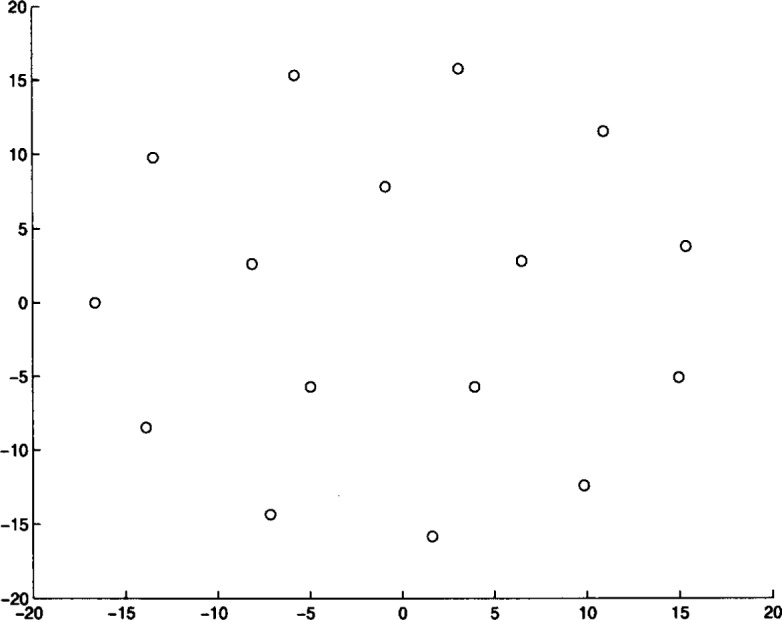
Optimal constellation for Cauchy noise, *M* = 16, sine-cosine basis.

**Fig. 8:**
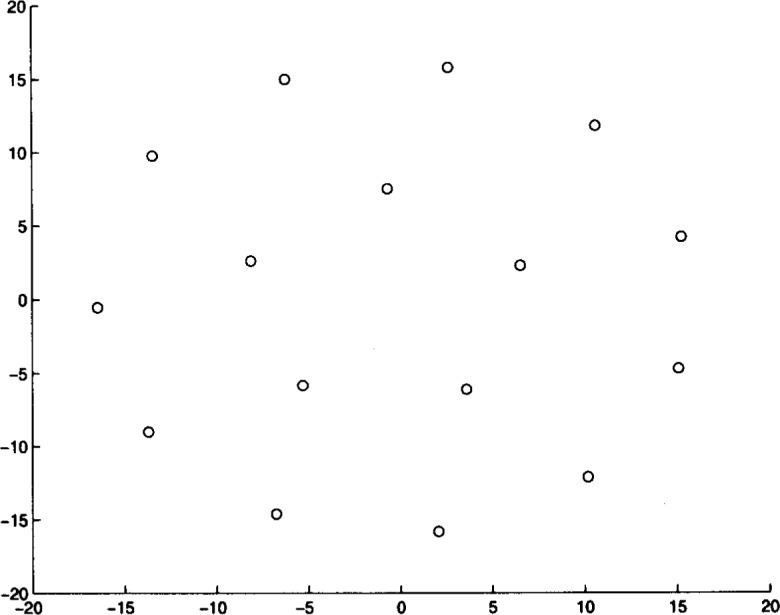
Optimal constellation for Hyperbolic Secant noise, *M* = 16, sine-cosine basis.

**Table 1:** Noise distributions and the associated KL distance function

Noise distribution	*p_N_*(*τ*)	*K_N_*(*δ*)
Gaussian	$\frac{1}{\sqrt{2\pi \sigma^{2}}}\exp\left(\frac{-\tau^{2}}{2\sigma^{2}}\right)$	$\frac{\delta^{2}}{2\sigma^{2}}$
Laplacian	$\frac{1}{\sqrt{2\pi \sigma^{2}}}\exp\left(\frac{-\sqrt{2}\cdot |\tau |}{\sigma }\right)$	$\frac{\sqrt{2}\cdot |\delta |}{\sigma }+\exp\left(\frac{-\sqrt{2}\cdot |\delta |}{\sigma }\right)-1$
Hyperbolic Secant	$\frac{1}{2\sigma }\text{sech}\left(\frac{\pi \tau }{2\sigma }\right)$	$-2\ln\left(\text{sech}\left(\frac{\pi \delta }{4\sigma }\right)\right)$
Generalized Gaussian	$\frac{1}{2\Gamma (5/4)a}\exp\left(\frac{-\tau^{4}}{a^{4}}\right)$	$\frac{\Gamma^{2}(3/4)}{\Gamma^{2}(1/4)}\left(6\frac{\delta^{2}}{\sigma^{2}}+\frac{\delta^{2}}{\sigma^{4}}\right)$
Cauchy	$\frac{1}{\pi \sigma (1+{\left(\tau /\sigma \right)}^{2})}$	$\ln\left(1+\frac{\delta^{2}}{4\sigma^{2}}\right)$

**Table 2:** Number of successful runs for **VF02AD** out of 20 trials

Noise distribution	sine-sine (*ϵ_r_* = 0)	sine-cosine (*ϵ_r_* = 0)	sine-cosine (*ϵ_r_* = 10^−6^)
Gaussian	4(1)	0	1(0)
Laplacian	6(1)	0	1(0)
Hyperbolic Secant	5(1)	0	0
Generalized Gaussian	6(2)	0	0
Cauchy	2(1)	0	0

**Table 3:** Number of successful runs of *BKT* SQP algorithm out of 20 trials

Noise distribution	sine-sine (*ϵ_r_* = 0)	sine-cosine (*ϵ_r_* = 0)	sine-cosine (*ϵ_r_* = 10^−6^)
Gaussian	20(8)	18(2)	18(1)
Laplacian	20(4)	20(3)	19(5)
Hyperbolic Secant	20(5)	20(1)	20(4)
Generalized Gaussian	20(4)	18(1)	20(1)
Cauchy	20(2)	19(1)	20(3)

**Table 4:** Optimal computed values for signal set design with *M* = 8

Noise	Basis	*F*(α*)
Gaussian	sine-sine	−69.793
	sine-cosine	−97.551
Laplacian	sine-sine	−63.122
	sine-cosine	−84.463
Hyperbolic Secant	sine-sine	−61.093
	sine-cosine	−83.196
Generalized Gaussian	sine-sine	−189.09
	sine-cosine	−264.18
Cauchy	sine-sine	−22.731
	sine-cosine	−30.673

**Table 5:** Optimal computed values for signal set design with *M* = 16

Noise	Basis	*F*(α*)
Gaussian	sine-sine	−29.314
	sine-cosine	−39.742
Laplacian	sine-sine	−32.370
	sine-cosine	−44.076
Hyperbolic Secant	sine-sine	−29.577
	sine-cosine	−40.500
Generalized Gaussian	sine-sine	−57.829
	sine-cosine	−76.138
Cauchy	sine-sine	−11.426
	sine-cosine	−15.688

## Footnotes

1 A major iteration is defined here as on solution of *PES*(*ρ*) and one update of *ρ*.

2 Certain commercial equipment, instruments, or materials are identified in this paper to foster understanding. Such identification does not imply recommendation or endorsement by the National Institute of Standards and Technology, nor does it imply that the materials or equipment identified are necessarily the best available for the purpose.

## References

[1] Boender CGE, Romeijn HE, Horst Reiner, Pardalos Panos (1995). Stochastic methods. Handbook of Global Optimization.

[2] Boggs PT, Kearsley AJ, Tolle JW (1999). A global convergence analysis of an algorithm for large-scale nonlinear optimization problems. SIAM J Opt.

[3] Boggs PT, Kearsley AJ, Tolle JW (1999). A practical algorithm for general large scale nonlinear optimization problems. SIAM J Opt.

[4] Boggs PT, Tolle JW (1996). Sequential quadratic programming. Acta Numerica.

[5] Foschini GJ, Gitlin RD, Weinstein SB (1974). Optimization or two-dimensional signal constellations in the presence of Gaussian noise. IEEE Trans on Comm.

[6] Gill PE, Murray W, Saunders MA (1997). SNOPT: An SQP algorithm for large-scale constrained optimization. Technical Report Report SOL 97-0.

[7] Gockenbach MS, Kearsley AJ (1999). Optimal signal sets for non-Gaussian detectors. SIAM J Opt.

[8] Gockenbach MS, Kearsley AJ, Symes WW (1997). An infeasible point method for minimizing the Lennard-Jones potential. Comp Opt Appl.

[9] Herskovits JN, Carvalho LAV, Bensoussan A, Lions JL (1986). A successive quadratic programming based feasible directions algorithm.

[10] Johnson DH, Orsak GC (1993). Relation of signal set choice to the performance of optimal non-Gaussian detectors. IEEE Trans on Comm.

[11] Rinooy Kan AHG, Timmer GT (1987). Stochastic global optimization methods; part II: multi-level methods. Math Prog.

[12] Kearsley AJ (1996). The use of optimization techniques in the solution of partial differential equations from science and engineering. Technical Report & PhD Thesis.

[13] Kiwiel KC (1983). A phase I—phase II method for inequality constrained minimax problems. Cont Cyb.

[14] Lawrence CT, Zhou JL, Tits AL User’s Guide for CFSQP Version 2.4: A C Code for Solving (Large Scale) Constrained Nonlinear (Minimax) Optimization Problems, Generating Iterates Satisfying All Inequality Constraints, 1986. ISR TR-94-16r1.

[15] Harwell Subroutine Library (1985). Library Reference Manual.

[16] Panier ER, Tits AL, Bensoussan A, Lions JL (1986). A superlinearly convergent method of feasible directions for optimization problems arising in the design of engineering systems.

[17] Panier ER, Tits AL (1987). A superlinearly convergent feasible method for the solution of inequality constrained optimization problems. SIAM J Con Opt.

[18] Panier ER, Tits AL (1993). On combining feasibility, descent and superlinear convergence in inequality constrained optimization. Math Prog.

[19] Polak E (1971). Computational Methods in Optimization.

[20] Poor HV (1994). An Introduction to Signal Detection and Estimation.

[21] Powell MJD, Watson GA (1978). A fast algorithm for nonlinearly constrained optimization calculations. Numerical Analysis, Dundee, 1977.

[22] Proakis JG (1989). Digital Communications.

[23] Rustem B, Nguyen Q (1998). An algorithm for the inequality-constrained discrete mini-max problem. SIAM J Opt.

[24] Spellucci P (1998). A new technique for inconsistent QP problems in the SQP method. Math of Oper Res.

[25] Zhou J (1992). Fast, Globally Convergent Optimization Algorithms, with Applications to Engineering System Design. PhD thesis.

[26] Zhou JL, Tits AL (1993). Nonmonotone line search for mini-max problems. J Optim Theory Appl.

